# Inversion of a part of the numerator relationship matrix using pedigree information

**DOI:** 10.1186/1297-9686-45-45

**Published:** 2013-12-06

**Authors:** Pierre Faux, Nicolas Gengler

**Affiliations:** 1 Animal Science Unit, Gembloux Agro-Bio Tech, University of Liège, Passage des Déportés, 2, 5030 Gembloux, Belgium

## Abstract

**Background:**

In recent theoretical developments, the information available (e.g. genotypes) divides the original population into two groups: animals with this information (*selected* animals) and animals without this information (*excluded* animals). These developments require inversion of the part of the pedigree-based numerator relationship matrix that describes the genetic covariance between *selected* animals (**A**_22_). Our main objective was to propose and evaluate methodology that takes advantage of any potential sparsity in the inverse of **A**_22_ in order to reduce the computing time required for its inversion. This potential sparsity is brought out by searching the pedigree for dependencies between the *selected* animals. Jointly, we expected distant ancestors to provide relationship ties that increase the density of matrix **A**_22_ but that their effect on $A_{{}_{22}}^{-1}$

might be minor. This hypothesis was also tested.

**Methods:**

The inverse of **A**_22_ can be computed from the inverse of the triangular factor (**T**^-1^) obtained by Cholesky root-free decomposition of **A**_22_. We propose an algorithm that sets up the sparsity pattern of **T**^-1^ using pedigree information. This algorithm provides positions of the elements of **T**^-1^ worth to be computed (i.e. different from zero). A recursive computation of $A_{{}_{22}}^{-1}$ is then achieved with or without information on the sparsity pattern and time required for each computation was recorded. For three numbers of *selected* animals (4000; 8000 and 12 000), **A**_22_ was computed using different pedigree extractions and the closeness of the resulting $A_{{}_{22}}^{-1}$ to the inverse computed using the fully extracted pedigree was measured by an appropriate norm.

**Results:**

The use of prior information on the sparsity of **T**^-1^ decreased the computing time for inversion by a factor of 1.73 on average. Computational issues and practical uses of the different algorithms were discussed. Cases involving more than 12 000 *selected* animals were considered. Inclusion of 10 generations was determined to be sufficient when computing **A**_22_.

**Conclusions:**

Depending on the size and structure of the *selected* sub-population, gains in time to compute $A_{{}_{22}}^{-1}$ are possible and these gains may increase as the number of *selected* animals increases. Given the sequential nature of most computational steps, the proposed algorithm can benefit from optimization and may be convenient for genomic evaluations.

## Background

For a population of *n* animals, the numerator relationship matrix (**A**), is an *n*-by-*n* matrix with the following properties:

(1) *a*_*ij*_ is the numerator relationship coefficient between two animals *i* and *j* among *n*, as defined by Wright [1];

(2) diagonal element *a*_*ij*_ is equal to 1 + *F*_*i*_, where *F*_*i*_ is the inbreeding coefficient [1] of animal *i*;

(3) **A** is non-singular and symmetric: for two animals *i* and *j* among *n*, *a*_*ij*_ = *a*_*ji*_.

Because the numerator relationship matrix describes the additive similarity between animals, it is an important element explaining genetic (co)variances between animals and has numerous applications in the field of animal genetics, the most important one being its use in setting up the mixed model equations for estimation of breeding values [2].

In some situations, a particular type of information (genomic information, foreign genetic evaluation, phenotypes on a particular trait, etc.) is only available for some animals, which are selected for this particular purpose, while other animals are excluded. The original population can therefore be split into two sub-populations:

(1) a sub-population composed of animals called “*excluded”* hereafter;

(2) a sub-population composed of animals called “*selected”* hereafter.

Splitting the original population in this way leads to the following partition of **A**:

$$A=\left[\begin{array}{cc} A_{11} & A_{12}\\ A_{21} & A_{22} \end{array}\right].$$

The four blocks include the relationships between *excluded* animals (**A**_11_), between *excluded* and *selected* animals (**A**_12_ and **A**_21_) and between *selected* animals (**A**_22_).

Recent methodological developments in animal breeding require inversion of **A**_22_, for example for genotyped animals in the context of genomic prediction using a single-step procedure [3-5]. Another example concerns external animals when integrating foreign information into a local genetic evaluation [6]. It is also noteworthy that the pedigree-based relationship matrix **A**_22_ and the genomic relationship matrix (**G**, [7]) show structural similarities: both matrices express polygenic/genomic similarities among animals inherited from ancestors that are not represented in these matrices. Thus, the present research on **A**_22_ can be extended to genomic relationships in **G**.

Based on the original work by Henderson [8] on inversion of **A**, a general framework for the inversion of relationship matrices follows (see Appendix 1). Henderson outlined a method that is based on the root-free factorization of **A** and showed the high sparsity of the inverse triangular factor of **A**. An efficient use of this sparsity then allows direct computation of **A**^-1^ as a sum of individual contributions based on a chronological reading of the pedigree. Applying partitioned matrix theory, van Arendonk et al. [9] gave a general expression for the sum of individual contributions outlined by Henderson [8]: an additional row/column in **A** leads to updating its inverse by increasing the order by 1 and by summing the square of a very sparse vector to **A**^-1^. The very sparse vector is the corresponding row (below the diagonal) of the inverse triangular factor. All details on these developments are given in Appendix 1.

When required, the inverse of **A**_22_ is currently obtained by brutal inversion algorithms (e.g. generalized inverse algorithm). In these algorithms, any potential sparsity occurring in the matrix to invert or in its inverse is brought out by matrix computations. In contrast, the main objectives of this paper were to investigate how potential sparsity in the inverse triangular factor of **A**_22_ can be characterized using only the pedigree, thus without requiring matrix computations, and then use the sparsity pattern of the inverse triangular factor of **A**_22_ in the computation of its inverse. Whereas the structure of the inverse triangular factor is known for **A** (positions are given by the pedigree; values are *a priori* known), no information is available on the structure of the inverse triangular factor of **A**_22_, neither on the positions of non-zero elements nor on the values of these elements. Moreover, the inverse triangular factor of **A**_22_ may be close to dense. Therefore, we addressed our objective in the following five steps:

(1) inversion of **A**_22_ with an algorithm that uses the inverse triangular factor;

(2) development of an algorithm that uses pedigree information to find the positions of the non-zero elements (sparsity pattern) in the inverse triangular factor of **A**_22_;

(3) inversion of **A**_22_ with the algorithm of step (1) but restricting computations to the non-zero elements identified by the algorithm in step (2);

(4) assessment of the time reduction when computing the inverse as in step (3) instead of as in step (1);

(5) and evaluation of the effect of the number of generations in the pedigree used to compute **A**_22_, in order to reduce density of the inverse triangular factor.

## Methods

### Blockwise inversion of A_22_

For simplicity, we assume that we are working on the last *selected* animal, indexed as animal *n*. Similarly to inversion of **A** (see equation 1.6 in Appendix 1), assume that **A**_22_ is partitioned in a sub-matrix **Z**, of order (*n*-1), a (*n*-1)-long vector **y**, and a scalar *m* as:

(1) $$A_{22}=\left[\begin{array}{cc} Z & y\\ y\prime & m \end{array}\right]$$

Using blockwise inversion, $A_{22}^{-1}$ can be recursively computed using the following equation:

(2) $$A_{22}^{-1}=\left[\begin{array}{cc} Z^{-1} & 0\\ 0^{\prime } & 0 \end{array}\right]+\frac{1}{s}\cdot \left[\begin{array}{c} -Z^{-1}y\\ 1 \end{array}\right]\;\left[\begin{array}{cc} -y^{\prime }Z^{-1} & 1 \end{array}\right]$$

where *s* is a scalar equal to *m* - **y**′**Z**^- 1^**y**.

Computing **b** = **Z**^- 1^**y** and defining *α* = *s*^- 1^ simplifies equation (2) as follows:

(3) $$A_{22}^{-1}=\left[\begin{array}{cc} Z^{-1} & 0\\ 0^{\prime } & 0 \end{array}\right]+\alpha \left[\begin{array}{c} -b\\ 1 \end{array}\right]\cdot \left[\begin{array}{cc} -b^{\prime } & 1 \end{array}\right]$$

Similarly, as for **A** (see Appendix 1), there is a link between vector **b** and the root-free Cholesky factorization of **A**_22_ (**A**_22_ = **TDT**′), in that –**b**′ corresponds to the last row of the inverse triangular factor of **A**_22_ (**T**^-1^).

Equation (3) shows that $A_{22}^{-1}$ can be constructed recursively by adding a vector product to the previous result (**Z**^-1^). This recursive construction of $A_{22}^{-1}$ will be called “Algorithm A” and implies, from the second row to the last row, the computation of the whole vector **b**.

If an animal and its parents are all *selected*, vector **b** is as sparse as in the case of **A**, i.e. the only non-zero elements of **b** correspond to parents. Restricting computations to these elements, i.e. discarding computations involving elements that we know equal 0, results in saving computing time. Such a case is, however, highly trivial. In the next sections, we propose a method to deal with more complex cases.

### Contribution of *selected* animals to relationships in A_22_: characterizing the sparsity pattern of T^-1^

**Figure 1:**
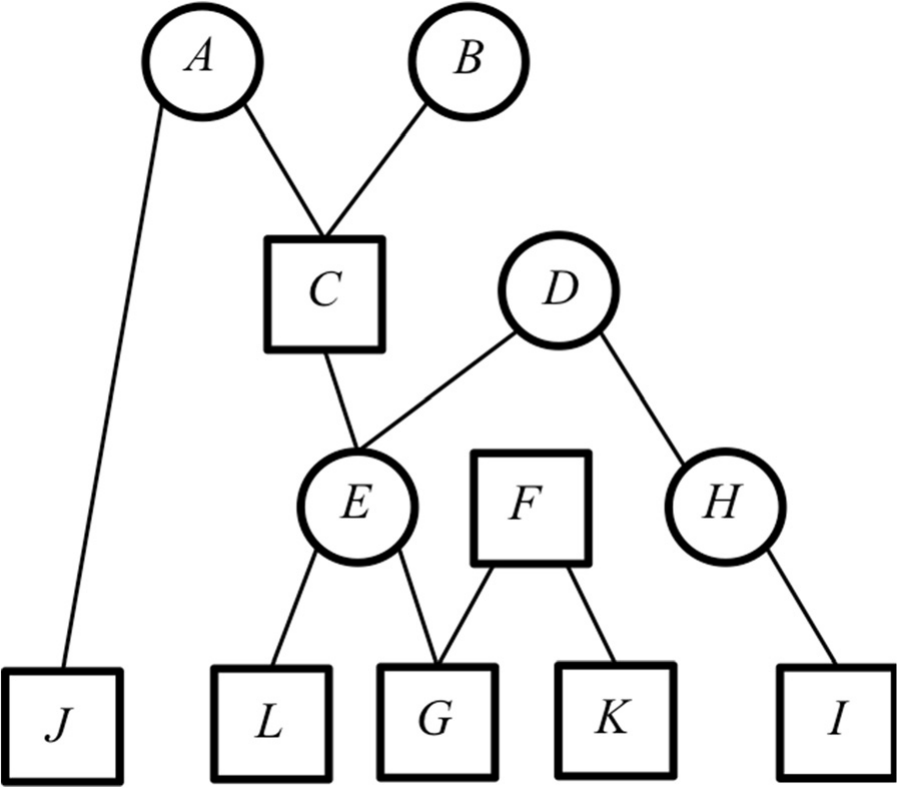
**Small example: a population of 12 animals.** Genealogical tree for a population of 12 animals, partitioned in sub-populations 1 (*excluded*, circle) and 2 (*selected*, square). Alphabetical order gives the birth order.

For animal *n*, vector **b** is the row of **T**^-1^ that spans from column 1 to column (*n*-1). By definition (**b** = **Z**^-1^**y** thus **Zb** = **y**), vector **b** contains the required coefficients to compute relationships (**y**) of animal *n* with the *n*-1 preceding animals from the relationships between those *n*-1 preceding animals (**Z**). In the case of **A**, only known parents of animal *n* are required to compute its relationships with the preceding animals. Therefore, only positions of known parents have a value different from zero in vector **b**. In the case of **A**_22_, some *selected* animals replace the parents if they are *excluded*: the value in **b** of these *selected* animals is different from 0, which means that they are needed to compute relationships between *selected* animals (**y**) from the relationships between all *selected* animals (**Z**). This can be illustrated by the example pedigree in Figure 1 and Tables 1 and 2, which specify**A**_22_ and **T**^-1^ for the example pedigree. Three cases are outlined and detailed in the following:

**Table 1:** **Matrix A**_**22 **_**for the example of Figure**1

	***C***	***F***	***G***	***I***	***J***	***K***	***L***
*C*	1.00		0.25		0.25		0.25
*F*		1.00	0.50			0.50	
*G*	0.25	0.50	1.00	0.06	0.06	0.25	0.25
*I*			0.06	1.00			0.06
*J*	0.25		0.06		1.00		0.06
*K*		0.50	0.25			1.00	
*L*	0.25		0.25	0.06	0.06		1.00

Empty cells are 0.

**Table 2:** **Inverse of the triangular factor (T**^-1^**) of A**_**22 **_**for the example of Figure**1

	***C***	***F***	***G***	***I***	***J***	***K***	***L***
*C*	1.00						
*F*		1.00					
*G*	-0.25	-0.50	1.00				
*I*	0.02	0.05	-0.09	1.00			
*J*	-0.25				1.00		
*K*		-0.50				1.00	
*L*	-0.18	0.13	-0.27	-0.05			1.00

Empty cells are 0.

(i) animal *G* has two known parents, *E* and *F*. Animal *E* is *excluded*; its parent *C* (grandparent of G) is thus required (**T**^- 1^_*GC*_ ≠ 0) to explain the relationship between *C* and *G* (**A**_22;*CG*_ = 0.25).

(ii) animal *K* has one known parent, *F*, that is also *selected*. Any relationship that *K* shares with other *selected* animals is necessarily and only explained by *F* ($\forall X\neq F,T_{\mathrm{KX}}^{-1}=0$).

(iii) animal *L* has one known parent, *E*, that is *excluded*. Its *selected* halfsib (*G*) and the *selected* parent of *G* (*F*, which is unrelated to *L*) are required, among others, to explain any relationship that *L* shares with other *selected* animals.

Animals that are required to explain relationships of a given *selected* animal with other *selected* animals will hereafter be denoted as the *contributors* of this *selected* animal. *Contributors* of a *selected* animal can be found by an exhaustive search of *selected* animals that replace any *excluded* parent of the *selected* animal. Their determination uses the pedigree and returns which elements of **b** (and thereby of **T**^-1^) are worth computing because they are expected to be non-zero. By subtraction, we obtain which elements are zeros, which is referred to as the “sparsity pattern” of **T**^-1^ in the following. In the next sub-section, we propose a heuristic algorithm that streamlines the determination of the sparsity pattern of **T**^-1^. Similar methodologies [10,11] have been developed for the triangular factor of a symmetric-positive definite matrix rather than the inverse of the triangular factor.

### An algorithm to set up the sparsity pattern

Our proposed heuristic algorithm to set up the sparsity pattern of the inverse triangular factor of **A**_22_ (see pseudo-code below) requires two inputs: the pedigree (of length *n*_0_, renumbered and ordered: parents precede progeny) and the subpopulation to which any animal belongs: *excluded* (population status is 1) or *selected* (population status is 2). The purpose of the algorithm is to complete two vectors of variable length for any animal *i*. The first vector (**r**_(*i*)_) contains references to *excluded* parents of animal *i*. The second vector (**c**_(*i*)_) contains *selected contributors* of animal *i*. The positions of non-zeros in the i-th row in **T**^-1^ (sparsity pattern) includes any position of the *i*-th row that is listed in **c**_(*i*)_.

Initialize a vector **x** as the integer sequence from 1 to *n*_0_.

For any animal *i* in the whole population (*i* goes from 1 to *n*_0_),

(0) initialize two vectors **c**_(*i*)_ and **r**_(*i*)_ as empty vectors

(1) if the status of animal *i* is 2, then append element *i* to **c**_(*i*)_; or else if the status of animal *i* is 1, append element *i* to **r**_(*i*)_

(2) if the sire *s* of animal *i* is known and its status is 2, then append element *s* to **c**_(*i*)_; or else if *s* is known but its status is 1, append vector **r**_(*s*)_ to **r**_(*i*)_

(3) if the dam *d* of animal *i* is known and its status is 2, then append element *d* to **c**_(*i*)_; or else if *d* is known but its status is 1, append vector **r**_(*d*)_ to **r**_(*i*)_

(4) if the status of animal *i* is 2 and the vector **r**_(*i*)_ is not empty, then:or else if the status of animal *i* is 1 or if the vector **r**_(*i*)_ is empty, do nothing.

a. Select all elements of **x** that are at positions given in **r**_(*i*)_, remove duplicates and gather them in a temporary list **t**

b. for any element *k* in list **t**,

i. Append to **c**_(*i*)_ the elements of vector **c**_(*k*)_ not yet in **c**_(*i*)_

ii. Select elements of **x** that are equal to *k* and replace them by i;

If the whole population was *selected* (i.e. **A**_22_ = **A**, every animal has status 2), it can be easily deduced from the algorithm that only the animal itself (in step (1)) and its known sire and dam (in steps (2) and (3)) would enter vector **c**_(*i*)_. The corresponding **T**^-1^ would be highly sparse, as it is for **A**. This also means that if numerous parents are *selected*, then this algorithm is expected to run very fast.

An example of the use of this algorithm is given in the Results section.

### Use of the sparsity pattern in blockwise inversion of A_22_

The algorithm for blockwise inversion of **A**_22_ (Algorithm A, summarized in equation (3)) is modified to account for sparsity and will be called Algorithm B. For simplicity, we still consider the last *selected* animal (animal *n*). Algorithm B reduces computations to obtain **b** from **y** = **Zb** (equations 2 and 3) by three procedures, depending on the number (*k*) of elements in the corresponding vector **c**_(*n*)_ and the length of **b** (*n*-1).

The first procedure (called *EMPTY*) is used when *k* = 0 (**c**_(*n*)_ is empty). If so, only *α* is added to element $A_{22,\mathrm{nn}}^{-1}$. The value of *α* is just the inverse of **A**_22,*nn*_.

The second procedure (called *PROD*, for matrix PRODuct) is used when *k* is smaller than but relatively close to (*n*-1). In such a case, we perform a line-wise partition (equation (4)) of **b** and **Z**^-1^ between non-zeros (of subscript *u*) and null (subscript *v*) entries of **b** in order to avoid useless computations:

(4) $$\left[\begin{array}{c} b_{u}\\ b_{v} \end{array}\right]=\left[\begin{array}{c} b_{u}\\ 0 \end{array}\right]=\left[\begin{array}{c} Z^{u}\\ Z^{v} \end{array}\right]y\Rightarrow \;b_{u}=Z^{u}y$$

Since (*n*-1) is the number of elements in **b** and *k* the number of elements in **b**_*u*_, *k* dot products (of (*n*-1)-long vectors) would be performed instead of (*n*-1) dot products (of (*n*-1)-long vectors).

The third procedure (called *LS*, for Linear System of lower size) is used when *k* is much smaller than (*n*-1). In such a case, we extend the previous partition of **b** to a blockwise partition of **Z** and **y** (the non-zero and zero elements of **b** are respectively indexed by *u* and *v*):

(5) $$\left[\begin{array}{c} b_{u}\\ b_{v} \end{array}\right]={\left[\begin{array}{cc} Z_{\mathrm{uu}} & Z_{\mathrm{uv}}\\ Z_{\mathrm{vu}} & Z_{\mathrm{vv}} \end{array}\right]}^{-1}\left[\begin{array}{c} y_{u}\\ y_{v} \end{array}\right]$$

Then, applying partitioned matrix theory to equation (5) returns the following expressions for **b**_*u*_ and **b**_*v*_ (with $S_{Z}=Z_{\mathrm{vv}}-Z_{\mathrm{vu}}Z_{\mathrm{uu}}^{-1}Z_{\mathrm{uv}}$):

$$\left\{\begin{array}{l} b_{u}=Z_{\mathrm{uu}}^{-1}y_{u}\;+\;Z_{\mathrm{uu}}^{-1}Z_{\mathrm{uv}}S_{Z}^{-1}Z_{\mathrm{vu}}Z_{\mathrm{uu}}^{-1}y_{u}\;-\;Z_{\mathrm{uu}}^{-1}Z_{\mathrm{uv}}S_{Z}^{-1}y_{v}\\ b_{v}=-S_{Z}^{-1}Z_{\mathrm{vu}}Z_{\mathrm{uu}}^{-1}y_{u}\;+\;S_{Z}^{-1}y_{v} \end{array}\right).$$

Vector **b**_*u*_ can be expressed in terms of **b**_*v*_ ($b_{u}=Z_{\mathrm{uu}}^{-1}y_{u}-Z_{\mathrm{uu}}^{-1}Z_{\mathrm{uv}}b_{v}$) and, since **b**_*v*_ is a vector of zeros, it comes that computing **b**_*u*_ shrinks to compute only $Z_{\mathrm{uu}}^{-1}y_{u}$. In other words, the linear system **Zb** = **y** is replaced by a linear system of lower size **Z**_*uu*_**b**_*u*_ = **y**_*u*_, and solving it is valuable only if the number of operations required to solve it is lower than the number of operations to achieve the product in procedure *PROD*. We chose the less expensive procedure (*PROD* or *LS*) by estimation of the number of expected floating-point multiplications.

### Experimental design for tests on real populations

In order to evaluate Algorithm B in comparison with regular inversion (Algorithm A), different **A**_22_ were computed on the basis of a real pedigree provided by the Luxembourg breeders society CONVIS. This pedigree includes dairy cows from Luxembourg with their ancestors tracing back up to 24 generations and contains 387 499 animals. Statistics of the pedigree data are Table 3.

**Table 3:** Statistics of the population used (dairy cows from Luxembourg)

**Total number of animals**	**387 499**
Number of cows	366 773
Number of bulls	20 726
**Number of animals by birth year class:**	
Before 1950	5441
From 1950 to 1974	24 577
From 1975 to 1999	229 016
From 2000 to 2012	128 465
**Maximum number of generations of pedigree**	39
**Average number of generations**^**1 **^**for animals in different birth year classes:**	
Before 1950	3.28
From 1950 to 1974	6.49
From 1975 to 1999	19.03
From 2000 to 2012	25.11
**Pedigree completeness: number of animals with (% of the pedigree):**	
Both parents unknown:	70 167 (18.1%)
Dam known, sire unknown	69 721 (18.0%)
Sire known, dam unknown	17 141 (4.4%)
Both parents known	230 470 (59.5%)

^1^For a given animal, the number of generations is computed as the number of generations between this animal and its most distant ancestor.

*Selected* sub-populations of three sizes (4000, 8000 and 12 000 animals) were designed and are identified hereafter as the three size scenarios S4k, S8k and S12k. Animals of the selected sub-populations were randomly chosen from a pool of animals born after 1999 (128 465 animals) on the assumption that only recent animals could be of interest (those being genotyped or in production).

**Figure 2:**
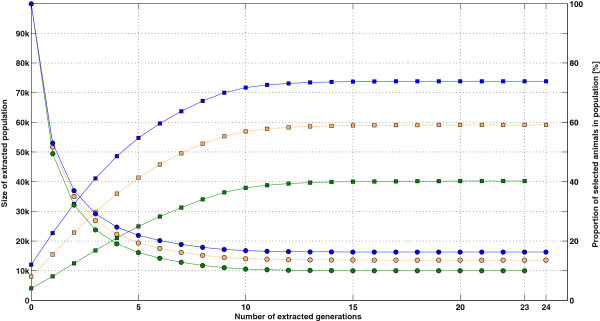
**Pedigree extraction facts.** Generation by generation extraction of the pedigree of the *selected* population for three size scenarios (green: S4k; orange: S8k; blue: S12k): number of extracted animals (■) and proportion of *selected* animals in the extracted population (●), expressed as a percentage. Extraction went up to 23 generations for scenario S4k and up to 24 generations for scenarios S8k and S12k.

Because a pedigree with a lower number of extracted generations is expected to provide a sparser **T**^-1^, the impact of the number of extracted generations was also evaluated for each size scenario. This enabled us to assess how many extracted generations were required in the pedigree to compute a $A_{22}^{-1}$ that is a sufficient approximation to the $A_{22}^{-1}$ computed using all available ancestors in the pedigree, which will be referred to as the “real inverse”. Extracting no animals other than *selected* animals refers to “generation 0”: the population is only made of *selected* animals. When extracting one generation of ancestors (“generation 1”), *excluded* parents enter the population. When extracting two generations of ancestors (“generation 2”), *excluded* grandparents also enter the population, and so on. Details on the number of animals extracted and the percentage of extraction after each generation, considered as the ratio between the number of animals in the population and the maximum number of animals available in the pedigree, are outlined in Figure 2.

Deviations from the real inverse were measured by the following norm: N=trA22g-A22f′A22g-A22ftrA22f′A22f, where $A_{22}^{\left(g\right)}$ is the inverse of **A**_22_ computed using *g* extracted generations and $A_{22}^{\left(f\right)}$ is the real inverse. This norm can be interpreted as the average difference between the value of any element of $A_{22}^{\left(g\right)}$ and its corresponding value in $A_{22}^{\left(f\right)}$. The two matrices are equal when *N* is equal to 0.

Matrix **A**_22_ was computed in two steps. Inbreeding coefficients were first computed for each size scenario and number of extracted generations. The average inbreeding coefficient was never greater than 1.23 % and the greatest inbreeding coefficient was 44.53%. Matrix **A**_22_ was then computed using the method of Colleau [12].

### Two test software programs

In order to evaluate potential gains in time when using Algorithm B instead of Algorithm A to invert **A**_22_, we developed two test programs in Fortran 95. The programs were neither optimized for speed, nor parallelized. Therefore, all comparisons have to be interpreted as relative figures.

The first program applies the recursive construction of the inverse, as outlined in Algorithm A (equations (2) and (3)). Potential null entries in **y** are checked to avoid useless computations when performing product **Z**^-1^**y**.

The second program restricts the same recursive construction of the inverse to non-zero elements by procedures *EMPTY*, *PROD* and *LS*. Potential null entries in **y** are also taken into account when performing the product **Z**^*u*^**y** (procedure *PROD*). The linear system **Z**_*uu*_**b**_*u*_ = **y**_*u*_ (procedure *LS*) is solved by factorization and by backward and forward substitutions.

For both programs, computing time was recorded using Fortran intrinsic subroutine CPU_TIME. For the program that uses Algorithm B, computing time includes the time required to determine the sparsity pattern. All computations and file storage were performed using double precision (15 digits). Each job was repeated 20 times on an Intel**®** Xeon**®** 64-bit processor (RAM: 8 Gb, cache size: 6 Mb, clock speed: 3 GHz).

## Results

### Characterizing the sparsity pattern: a numerical example

The algorithm to characterize the sparsity pattern was applied to the example pedigree of Figure 1 and specified in Table 4 (including animal status). The algorithm starts by initializing a vector **x** equal to [1, 2, 3, 4, 5, 6, 7, 8, 9, 10, 11, 12]. Then, we consecutively treat each animal depending on its status and the status of its parents.

**Table 4:** **Renumbered pedigree for the example of Figure**1

	***Number***	***Sire***	***Dam***	***Status***
*A*	1	-	-	1
*B*	2	-	-	1
*C*	3	1	2	2
*D*	4	-	-	1
*E*	5	3	4	1
*F*	6	-	-	2
*G*	7	6	5	2
*H*	8	-	4	1
*I*	9	8	-	2
*J*	10	1	-	2
*K*	11	6	-	2
*L*	12	-	5	2

Population status of the animal is given in a 4th column: 1 for *excluded*, 2 for *selected*.

Animal 1. Status 1 and unknown parents. Thus, **r**_(1)_ = [1], **c**_(1)_ = [-] and **x** = **x**.

Animal 2. Status 1 and unknown parents. Thus, **r**_(2)_ = [2], **c**_(2)_ = [-] and **x** = **x**.

Animal 3. Status 2 and known parents (1 and 2; both status 1). Thus, **c**_(3)_ = [3] and **r**_(3)_ = [1, 2]. The list of elements of **x** that match **r**_(3)_ is [1,2]. Then, **c**_(3)_ = [3, **c**_(1)_, **c**_(2)_] = [3] and any element of **x** equal to 1 or 2 is replaced by 3, returning **x** = [3, 3, 3, 4, 5, 6, 7, 8, 9, 10, 11, 12].

Animal 4. Status 1 and unknown parents. Thus, **r**_(4)_ = [4], **c**_(4)_ = [-] and **x** = **x**.

Animal 5. Status 1 and known parents (status 1 and 2). Thus, **c**_(5)_ = [3] and **r**_(5)_ = [5, **r**_(4)_] = [5, 4]. No list to set up because animal has status 1; **x** = **x**.

Animal 6. Status 2 and unknown parents. Thus, **r**_(6)_ = [-], **c**_(6)_ = [6] and **x** = **x**.

Animal 7. Status 2 and known parents (status 1 and 2). Thus, **c**_(7)_ = [7, 6] and **r**_(7)_ = [**r**_(5)_] = [5, 4]. The list of elements of **x** that match **r**_(7)_ is [4,5]. Then, **c**_(7)_ = [7, 6, **c**_(5)_, **c**_(4)_] = [7, 6, 3] and any element of **x** equal to 5 or 4 is replaced by 7, returning **x** = [3, 3, 3, 7, 7, 6, 7, 8, 9, 10, 11, 12].

Animal 8. Status 1 and one known parent (status 1). Thus, **r**_(8)_ = [8, **r**_(4)_] = [8, 4], **c**_(8)_ = [-] and **x** = **x**.

Animal 9. Status 2 and one known parent (status 1). Thus, **c**_(9)_ = [9] and **r**_(9)_ = [**r**_(8)_] = [8, 4]. The list of elements of **x** that match **r**_(9)_ is [7,8]. Then, **c**_(9)_ = [9, **c**_(8)_, **c**_(7)_] = [9, 7, 6, 3] and any element of **x** equal to 8 or 7 is replaced by 9, returning **x** = [3, 3, 3, 9, 9, 6, 9, 9, 9, 10, 11, 12]

Animal 10. Status 2 and one known parent (status 1). Thus, **c**_(10)_ = [10] and **r**_(10)_ = [**r**_(1)_] = [1]. The list of elements of **x** that match **r**_(10)_ is [3]. Then, **c**_(10)_ = [10, **c**_(3)_] = [10, 3] and any element of **x** equal to 3 is replaced by 10, returning **x** = [10, 10, 10, 9, 9, 6, 9, 9, 9, 10, 11, 12]

Animal 11. Animal has status 2 and has one known parent (status 2). Thus, **c**_(11)_ = [11, 6] and **r**_(11)_ = [-]. No list to set up because **r**_(11)_ is empty; **x** = **x**

Animal 12. Status 2 and one known parent (status 1). Thus, **c**_(12)_ = [12] and **r**_(12)_ = [**r**_(5)_] = [5, 4]. The list of elements of **x** that match **r**_(12)_ is [9]. Then, **c**_(12)_ = [12, **c**_(9)_] = [12, 9, 7, 6, 3] and any element of **x** equal to 9 is replaced by 12, returning **x** = [10, 10, 10, 12, 12, 12, 12, 12, 12, 10, 11, 12]

Vectors **c**_(*i*)_ of the *selected* animals (3, 6, 7, 9, 10, 11 and 12) contain the non-zero elements of **T**^-1^ (Table 5) and these match with **T**^-1^ in Table 2.

**Table 5:** **Sparsity pattern of T**^-1^**for the example of Figure**1

	***C***	***F***	***G***	***I***	***J***	***K***	***L***
*C*	X						
*F*		X					
*G*	X	X	X				
*I*	X	X	X	X			
*J*	X				X		
*K*		X				X	
*L*	X	X	X	X			X

x indicates non-zero entries.

### Effect of accounting for sparsity on CPU time for inversion of A_22_

**Figure 3:**
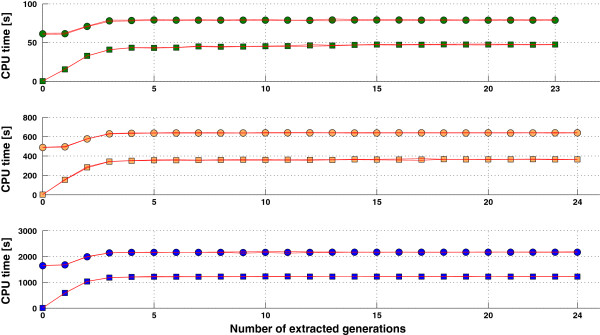
**CPU time required for inversion of A**_**22 **_**by two algorithms.** Elapsed CPU time required for inversion of **A**_22_ of three different sizes (green: 4000; orange: 8000; blue: 12000), computed using pedigrees with different numbers of extracted generations, by algorithms B (■) and A (●). Red lines show upper and lower confidence intervals (99%; 20 repetitions).

Algorithms A and B were both applied to the matrices created by different pedigree extractions of the three size scenarios. The elapsed CPU time results (averaged over 20 repetitions) are shown in Figure 3. Taking sparsity into account (Algorithm B) instead of using an inversion algorithm with cubic complexity (Algorithm A) reduced the elapsed CPU time for computing the inverse. For instance, the relative gains in computing speed of Algorithm B for the fully extracted pedigree were 1.67 faster for S4k, 1.75 faster for S8k, and 1.77 faster for S12k.

### Effect of the number of extracted generations on accuracy of $A_{22}^{-1}$

**Figure 4:**
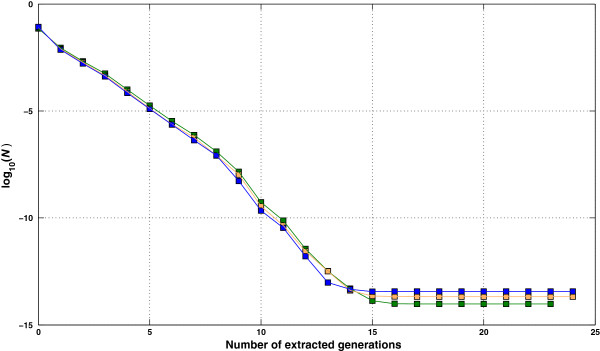
**Effect of the depth of the pedigree on**$A_{22}^{-1}$**.** Differences, as base-10 logarithm of the norm *N*, between $A_{22}^{-1}$ based on a pedigree with a limited number of extracted generations and $A_{22}^{-1}$ based on a fully extracted pedigree, for three size scenarios (green: S4k; orange: S8k; blue: S12k).

For each size scenario, **A**_22_ was computed using different numbers of extracted generations and the inverses were compared (Figure 4) to $A_{22}^{-1}$ computed using the fully extracted pedigree (after 23, 24 and 24 generations respectively for scenarios S4k, S8k and S12k) by computing the norm *N*. As shown in Figure 4, regardless of the size of the matrix, the norm stabilized after 14 generations to values less than 1E-13, which can be attributed to errors due to precision.

## Discussion

### Computation time required by the algorithm to characterize the sparsity pattern

**Figure 5:**
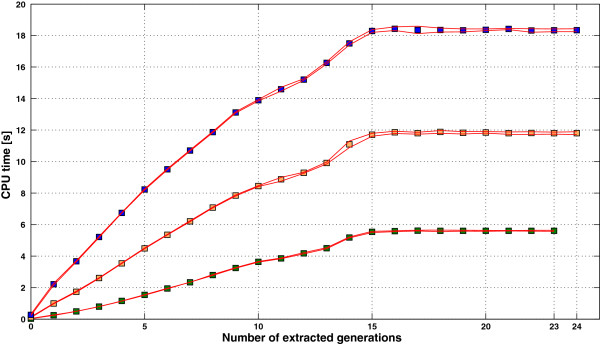
**CPU time required for determination of the sparsity pattern of T**^**-1**^**.** Elapsed CPU time required by the proposed algorithm for determination of the sparsity pattern of **T**^**-1**^, by number of extracted generations, for three size scenarios (green: S4k, orange: S8k and blue: S12k). Red lines show upper and lower confidence intervals (99%; 20 repetitions).

Figure 5 shows the elapsed CPU time (averaged over 20 repetitions) when running the proposed algorithm to determine the sparsity pattern of **T**^-1^ on populations with different numbers of *selected* animals (4000; 8000; 12 000) and that were extracted from several generations. The curves of the three size scenarios (S4k, S8k and S12k) presented a similar behavior. When the population consists only of *selected* animals (generation 0), the elapsed time was less than 1 second (S4k: 0.03 s, S8k: 0.11 s and S12k: 0.29 s). For this case, only non-zero entries occur for *selected* sires or dams of *selected* animals, *a fortiori* present in the pedigree. Then, elapsed CPU time increased linearly up to the 15th extracted generation, although at a different rate for the different size scenarios. Beyond that point, adding ancestors did not affect the elapsed time. These results have to be related with pedigree extraction (Figure 2): does it make sense to spend more time for additional generations? Almost all available ancestors have entered the population after extracting 10 generations (between 95-99% of the number of animals in the last extraction round). However, elapsed CPU time continued to increase at the same rate from generations 10 to 15. For instance, in scenario S12k, adding ~3% of the final population cost an additional ~4 seconds (or ~22% of the total elapsed time). The usefulness of this small group of remote ancestors for inversion of **A**_22_ is discussed hereafter (sub-section “*Number of generations to extract*”).

For the fully extracted population (after 23, 24 and 24 generations for scenarios S4k, S8k and S12k, respectively), there was a close-to-linear relationship between the size of the *selected* population and the elapsed CPU time (approximately 6 seconds for 4000 additional animals in the *selected* sub-population). The effective computational complexity of this algorithm is difficult to establish, however, because it mostly depends, first, on how the population was split (for instance, a *selected* sub-population that includes mainly a few lines or families would not contain that many *excluded* parents) and, secondly, on how the population is structured (depth of the pedigree, effective size of the population, average inbreeding). The embedded loop in the algorithm (step (4b) in the pseudo-code) is the main computational bottleneck and performs *k* iterations. In a population of *n*_0_ animals, if *k* is related to the two factors mentioned above (i.e. splitting and structure of the population), then the computing time required by the algorithm would behave as *n*_0_ · *k*, where *k* would be a case-specific factor. This agrees with the observations in Figure 5.

### Memory requirements of the algorithm to characterize the sparsity pattern

For a population of *n*_0_ animals with *n selected* animals, vectors **c**_(*i*)_ and **y**_(*i*)_ have the greatest RAM requirements. In our implementation, vector **y**_(*i*)_ stores few elements (positions of *excluded* ancestors) for all animals (thus ~ *n*_0_ integers). For *selected* animals, vector **c**_(*i*)_ stores non-zero positions and includes approximately $n\cdot \left(n\cdot \bar{d}\right)$ integers, where $\bar{d}$ is the average density of **T**^-1^ (number of non-zeros in the lower part of **T**^-1^ averaged by line). For *excluded* animals, **c**_(*i*)_ accounts for potential *selected* ancestors, therefore including approximately $\left(n_{0}-n\right)\cdot \bar{a}$ integers, where $\bar{a}$ is the average number of *selected* ancestors per *excluded* animal. Memory would thus be allocated for approximately $n^{2}\bar{d}+\left(n_{0}-n\right)\cdot \bar{a}$ integers. None of these integers may be declared as 3-byte integers when *n*_0_ is lower than 2^24^ (i.e. when pedigree contains less than 16.77 millions of animals).

### Use of the algorithm to characterize the sparsity pattern on greater populations

**Figure 6:**
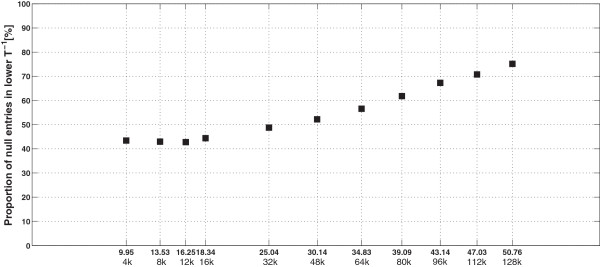
**Degree of sparsity of T**^**-1**^**.** Proportion of null entries in the lower triangular part of **T**^- 1^ for different proportions (%) and numbers (thousands of animals) of *selected* animals in an extracted population.

**Figure 7:**
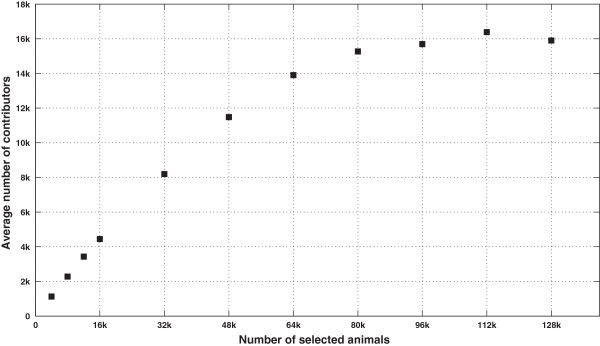
**Average number of *contributors*.** Average number of *contributors* by line of **T**^-1^, for different numbers of *selected* animals (in thousands of animals).

If additional animals are *selected*, then the proportion of *selected* animals in the population would likely increase. In fact these additional animals would either bring new *excluded* ancestors (case 1), share ancestors with already *selected* animals (case 2), or have no registered parents in the pedigree (case 3). The two last cases are expected to be more important as the number of *selected* animals increases. Therefore, matrix **T**^-1^ of such a population should get sparser. These expectations were confirmed by randomly picking animals from the pool of 128 465 animals born after 1999, simulating eight larger *selected* sub-populations of 16 000 up to 128 000 animals. Table 6 gives sizes and proportions of the *selected* sub-populations. Using a computer with higher memory resources (64 Gb of RAM), the sparsity pattern of these new situations was computed. Then, the degree of sparsity was assessed as the percentage of null entries in the lower triangular part of **T**^-1^ for these new situations, as well as for previous size scenarios. The results in Figure 6 show that the degree of sparsity remained the same for low percentages of *selected* animals in the population (lower than 20%), while the degree of sparsity increased linearly beyond approximately 20 k animals in these specific cases. The average degree of sparsity by number of *selected* animals corresponded to the average number of *contributors* for a given animal in a given size situation. Figure 7 shows that the average number of *contributors* was linearly related to the number of *selected* animals up to ~80 k *selected* animals, beyond which the average number of *contributors* was constant. We expected the average number of contributors to decrease as the number of *selected* animals increased. These new *selected* animals would then cover more of the relationships due to *excluded* animals. Note that the average number of contributors would be less than 2 if all animals were *selected* (i.e. **A**_22_ = **A**).

**Table 6:** **Populations extracted for different sets of *selected *** **animals**

**Number of *selected *** **animals**	**Size of the extracted population**	**Proportion of *selected *** **animals in the extracted population (%)**
4 000	40 196	9.95
8 000	59 120	13.53
12 000	73 864	16.25
16 000	87 237	18.34
32 000	127 809	25.04
48 000	159 259	30.14
64 000	183 750	34.83
80 000	204 637	39.09
96 000	222 546	43.14
112 000	238 130	47.03
128 000	252 147	50.76

### Computation time required by the algorithm for inversion of A_22_ using the sparsity pattern (Algorithm B)

**Figure 8:**
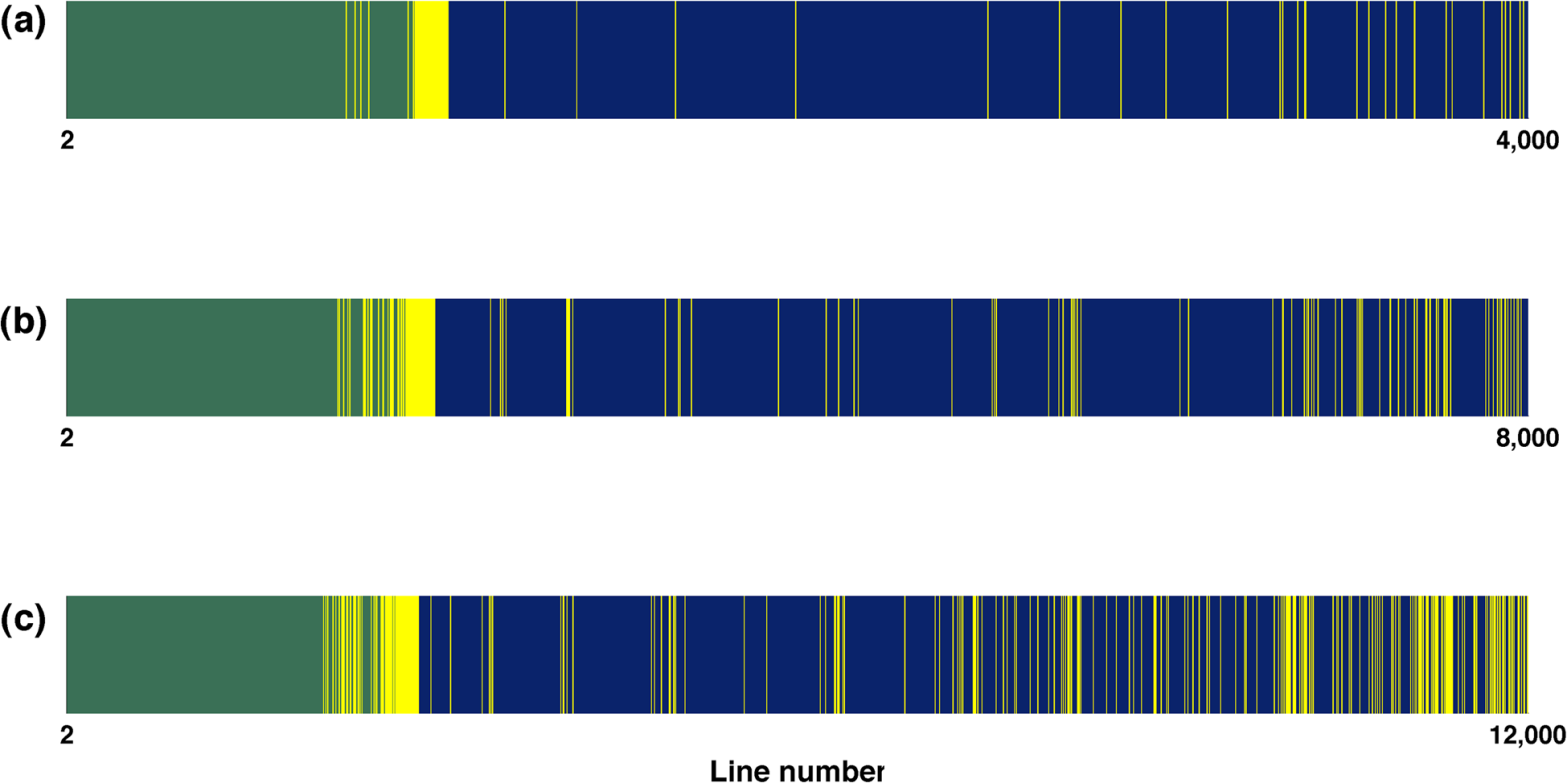
**Procedure choice when running algorithm B.** Procedure choice (green: *EMPTY*; yellow: *LS*; blue: *PROD*) when running algorithm B, along all line numbers of **T**^-1^, for inversion of matrix **A**_22_ with a fully extracted pedigree, for three size scenarios [**(a)**: S4k; **(b)**: S8k ; **(c)**: S12k].

When running Algorithm B, the procedure (*EMPTY*, *PROD* or *LS*) to compute vector **b** was chosen according to the estimated number of floating-point multiplications to be performed. A view of this choice along all (*n*-1) lines of **T**^-1^ is given in Figure 8 for each size scenario (**A**_22_ was always computed using a fully extracted pedigree). Due to prior reordering of the pedigree by generation, the first lines of **T**^-1^ correspond to founders (unrelated animals) and are thus empty. Procedure *LS* occurred less than procedures *EMPTY* and *PROD* but was evenly distributed among line numbers, particularly for scenario S12k.

**Figure 9:**
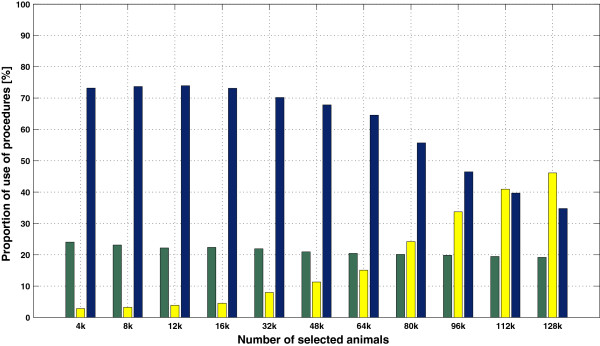
**Proportional use of different procedures in algorithm B.** Proportional (%) use of the three procedures in algorithm B (green: *EMPTY*; yellow: *LS*; blue: *PROD*), for different numbers of *selected* animals (in thousands of animals).

Considering Algorithm B led to estimation of the computational complexities based on the expected number of floating-point multiplications involved in the different tasks achieved by Algorithm B, as specified in Table 7. Total complexity is detailed for treatment of one line and for treatment of one full matrix of order *n* in Table 7, where treatment refers to all tasks to be performed, i.e. computing **b** and adding **bb**′ to the previous inverse. If *k* (average number of *contributors*) is considered as independent of *n*, the most complex term is *O*(*n*^2^ · *k*), which is required when using the *PROD* procedure (proportion *p*_*P*_ of the total). The *PROD* procedure is used less frequently for greater matrices (see Figures 8 and 9 beyond 80 k animals). Treating *k* as independent of *n* is also a more reasonable assumption for greater matrices (Figure 7), since *k* is undoubtedly related to *n* for smaller matrices. The total complexity for a matrix of order *n* becomes:

$$\begin{array}{l} {\left(\bar{d}\right)}^{3}p_{L}\;O(n^{4})+\left[\bar{d}\;p_{P}+{\left(\bar{d}\right)}^{2}\left(p_{L}+p_{P}\right)\right]\cdot O(n^{3})\\ +\bar{d}\;p_{L}O\left(n^{2}\right)+p_{E}O(n) \end{array},$$

where $\bar{d}$ represents the average density of the matrix. The most complex term $\left({\left(\bar{d}\right)}^{3}p_{L}O\left(n^{4}\right)\right)$ is tempered by two very low coefficients: the proportion of times the *LS* procedure is used (*p*_*L*_), which may be very low for small matrices (Figure 9), and the cube of the average density $\left(\bar{d}\right)$, which was lower than 0.5 in our examples (Figure 6) for matrices of order beyond 32 000. Thus, Algorithm B seems more suitable for large matrices than for small matrices, regardless of whether there is dependence between *n* and *k* or not.

**Table 7:** **Estimated computational complexity**^**1 **^**of Algorithm B**

**Procedure**	**Complexity for line *i***	**Proportion**	**Complexity on *n *** **lines**
*EMPTY*	1	*p*_E_	*p*_*E*_ · *O*(*n*)
*LS*	*O*(*k*^3^) + *O*(*k*^2^) + *O*(*k*)	*p*_L_	*p*_*P*_ · [*O*(*n* · *k*^3^) + *O*(*n* · *k*^2^) + *O*(*n* · *k*)]
*PROD*	*O*(*k*^2^) + *O*(*k* · *i*)	*p*_P_	*p*_*P*_ · [*O*(*n* · *k*^2^) + *O*(*n*^2^ · *k*)]
**Total**	$$\begin{array}{l} O\left(k^{3}\right)+O(k^{2})\\ +O\left(k\cdot i\right)+O(k) \end{array}$$	1	$$\begin{array}{l} p_{P}\cdot O\left(n^{2}\cdot k\right)+p_{L}\cdot O(n\cdot k^{3})+(p_{L}+p_{P})\cdot O(n\cdot k^{2})\\ +p_{L}\cdot O\left(n\cdot k\right)+p_{E}\cdot O(n) \end{array}$$

Matrix is of order *n* and average number of *contributors* is *k*;

^1^computational complexity is assessed as the expected number of floating-point multiplications to be performed.

The issue of numerical stability was also addressed. When using procedure *PROD*, the result of the previous iteration was used in the current iteration through *α* and **b**. Accumulating errors could lead to instabilities and/or divergences. However, in *LS* procedure*,* the result of the previous iteration does not affect the **b** that is computed. Choosing the *LS* procedure at regular intervals among iterations using the *PROD* procedure (see Figure 8) stops the accumulation of errors that could have resulted from continuously choosing the *PROD* procedure. Therefore, interlacing choices for both procedures is a good way to prevent numerical instability. Independence between iterations also allows procedure *LS* to parallelized.

### Memory requirements of the algorithm for inversion of A_22_ using sparsity pattern (Algorithm B)

Algorithm B requires allocation of more than twice the RAM than Algorithm A because it cannot store the results of the inversion in the input matrix. This is due to procedure *LS* working on different parts of **A**_22_. However, since elements that are required for *LS* are identified when determining the sparsity pattern, they could be stored separately in order to reduce the amount of RAM required. For that reason, sparsity patterns should be established prior to computation of **A**_22_ to determine which relationships are worth being computed.

### Number of generations to extract

The depth of the pedigree to be used for instance in genetic evaluations, is still a question of debate, and often moderately deep pedigrees are used, especially when only recent data is analyzed.

Results in Figure 4 suggest that pedigree from a limited number of generations (5 to 10) is sufficient to compute $A_{22}^{-1}$ with reasonable accuracy. The explanation is that distant ancestors do not greatly enhance a relationship. For instance, a common ancestor to animals *i* and *j* that enters the pedigree after *g* extracted generations and that is older than any selected animal, can only add up to 2^- 2*g*^ to the value of the relationship between *i* and *j*. In generation *g, i* and *j* can have a maximum of 2^*g*^ common ancestors*.* Therefore, extracting an additional generation can increase the relationship between *i* and *j* by only up to *δ* = 2^- *g*^. Regardless of the number of animals added to the pedigree when extracting generation 10, the maximum change brought to any relationship reduces to less than 0.001, which would have a minor effect on the inverse scale, as confirmed by Figure 4.

However, computing time required for determination of the sparsity pattern increases linearly after 10 generations (Figure 5). Thus, limiting extraction of pedigree to 10 generations appears to be a good balance between taking into account relationships due to distant ancestors and computing time. Applying a similar study to pedigree extractions for routine genetic evaluations would be meaningful and may lead us to consider extracting a number of generations instead of a birth year limit, which is current common practice.

### Practical use in a genomic background

For genomic evaluations, two specific situations where $A_{22}^{-1}$ is needed may require the use of Algorithm B. First, as explained above and shown in equation (3), the inverse of the matrix is computed recursively by adding a block specific to the current animal to the previous inverse. At each genomic evaluation, $A_{22}^{-1}$ could therefore be stored in a file and reused at the next evaluation cycle. At each evaluation, the matrix would be enhanced by adding newly genotyped animals. However, this approach has some limits:

(1) Animals have to be listed by generation order and only animals younger than those already genotyped can be added because older animals may cause changes in the sparsity pattern. This could be easily implemented in a cattle breed such as Holstein, where only few animals are key ancestors of the breed.

(2) The resulting file may be large but this could be reduced by sparse storage approaches.

Meyer et al. [13] recently applied a similar methodology for computation of the inverse of the genomic relationship matrix (**G**): their methodology also updates the previous inverse of **G**, necessitating its storage on disk from an evaluation to the next one.

Secondly, when using a pedigree of only one extracted generation, which contains genotyped animals and their ungenotyped parents, inversion of **A**_22_ is even faster (Figure 6) and the inverse seems to be a reasonable approximation of $A_{22}^{-1}$ computed with a full extracted pedigree (see Figure 4 and Discussion here above). Such a fair approximation of $A_{22}^{-1}$ may be useful as a preconditioner to solve **A**_22_**x** = **v**, for instance, as required in the iterative solution of MME of single-step genomic BLUP (best linear unbiased prediction) proposed by Legarra and Ducrocq [14].

### Current limits

The algorithm to determine the sparsity pattern of the inverse triangular factor of **A**_22_ is obviously useful only in inversion algorithms that use the inverse triangular factor. For other inversion algorithms, the algorithm to determine the sparsity pattern should not be useful.

Inversion algorithms that use the inverse triangular factor are useful in certain cases (e.g., for updating an inverted matrix or for obtaining quick approximations), but they would be less efficient, in terms of computing time, for the single purpose of inversion. The time required by Algorithms A and B was compared with the time required by subroutine “dkmxhf.f90” (K. Meyer, University of New England, Australia), which is a regular and efficient inversion algorithm. For inversion of the three different orders of **A**_22_ (4000, 8000 and 12 000), computing times of dkmxhf.f90 were lower than computing times obtained with Algorithm A and similar to those obtained with Algorithm B (accounting for sparsity). For small numbers of extracted generations, computing times were slightly lower for Algorithm B than dkmxhf.f90, but were greater when greater numbers of generations were extracted. However, the computing speed of Algorithm B can benefit from several optimizations (e.g., parallelization of the *LS* procedure and use of specific libraries for matrix products).

For computational ease, a small population (less than 1 million animals) was used in this study. Gains in computing time have to be tested for other sizes of population. This study was also restricted to only one population by size scenario and used repetitions (20) of the algorithm on the same data. Use of a Holstein population may also be criticized because although the average computed inbreeding was never greater than 1.23%, such a population has few key ancestors. Having the key ancestors in the *selected* sub-population might avoid density, because they would be *contributors* of many other *selected* animals.

## Conclusions

The determination of the sparsity pattern of **T**^-1^ using pedigree information is a prior step that allows gains in computing time for inversion based on the use of **T**^-1^. This allowed the computing time for inversion of matrices of three different sizes (4000, 8000 and 12 000 *selected* animals) to be reduced by a factor 1.73 on average. Gains in computing time are expected to be higher if the number of *selected* animals exceeds 80 000. Memory requirements for inversion of such a matrix would increase and the algorithm would become numerically more stable, since the *LS* procedure would become more important than the *PROD* procedure. Moreover, computation of the inverse by a recursive method may be very helpful in the case of genomic prediction, where a new batch of younger *selected* animals at each upcoming evaluation must be added to the previous inverse matrix already computed.

The results on the number of pedigree generations required for the *selected* animals suggest that no more than 14 generations should be extracted. If the working precision is less than 15 digits, this can even be reduced. A good balance between computing time for determination of the sparsity pattern and accuracy may be achieved with 10 extracted generations.

## Appendix

### Appendix 1: Inversion of the numerator relationship matrix using the inverse triangular factor

The numerator relationship matrix (**A**) can be factorized as

(1.1) $$A=\mathrm{TD}T^{\prime }.$$

Henderson [8] proposed a recursion rule to compute the triangular factor **T**:

(1.2) $$T_{\left(i\right)}=\left[\begin{array}{cc} T_{\left(i-1\right)} & 0\\ b_{\left(i\right)}\prime \;T_{\left(i-1\right)} & 1 \end{array}\right]$$

In equation (1.2), **T**_(*i*-1)_ and **T**_(*i*)_ are two matrices of respective sizes (*i*-1) and *i*. They refer **T** computed after, respectively, (*i*-1) and *i* recursions. Vector **b**_(*i*)_ is a vector of parental contributions: it summarizes the linear dependency between parents and offspring. This vector is null except on positions corresponding to parents of *i* where it is equal to 0.5. Henderson [8] also showed that the inverse triangular factor (**T**^-1^) only contains three different values: 0, 1and -0.5, since it is obtained by triangular matrix inversion (equation 1.3). The elements of the diagonal are equal to 1 and the lower off-diagonal elements are equal to the vector $-b_{\left(i\right)}^{\prime }$ corresponding to the *i*^th^ animal; they contain thus only 0 and -0.5 elements.

(1.3) $$T_{\left(i\right)}^{-1}=\left[\begin{array}{cc} T_{\left(i-1\right)}^{-1} & 0\\ -b_{\left(i\right)}^{\prime } & 1 \end{array}\right]$$

Besides **T**, the diagonal matrix **D** is computed one element at a time according to Henderson [8] and Quaas [15]. At the *i*^th^ recursion **D**_(*i*)_ has the form:

(1.4) $$D_{\left(i\right)}=\left[\begin{array}{cc} D_{\left(i-1\right)} & 0\\ 0^{\prime } & d_{\mathrm{ii}} \end{array}\right]$$

Replacing equations (1.2) and (1.4) in (1.1) shows that the recursion rule for computation of **T** is actually identical to that of the tabular method (equation 1.5.3, Emik and Terril [16]; Henderson [8]), since it computes the last below-diagonal row in **A**_(*i*)_ as a linear combination of rows in **A**_(*i*-1)_.

(1.5.1) $$A_{\left(i\right)}=T_{\left(i\right)}D_{\left(i\right)}{T_{\left(i\right)}}^{\prime }$$

(1.5.2) $$=\left[\begin{array}{cc} T_{\left(i-1\right)}D_{\left(i-1\right)}T_{\left(i-1\right)}^{\prime } & T_{\left(i-1\right)}D_{\left(i-1\right)}T_{\left(i-1\right)}^{\prime }b_{\left(i\right)}\\ b_{\left(i\right)}^{\prime }T_{\left(i-1\right)}D_{\left(i-1\right)}T_{\left(i-1\right)}^{\prime } & b_{\left(i\right)}^{\prime }T_{\left(i-1\right)}D_{\left(i-1\right)}T_{\left(i-1\right)}^{\prime }b_{\left(i\right)}+d_{\mathrm{ii}} \end{array}\right]$$

(1.5.3) $$=\left[\begin{array}{cc} A_{\left(i-1\right)} & A_{\left(i-1\right)}b_{\left(i\right)}\\ b_{\left(i\right)}^{\prime }A_{\left(i-1\right)} & b_{\left(i\right)}^{\prime }A_{\left(i-1\right)}b_{\left(i\right)}+d_{\mathrm{ii}} \end{array}\right]$$

Replacing $b_{\left(i\right)}^{\prime }A_{\left(i-1\right)}b_{\left(i\right)}+d_{\mathrm{ii}}$ in equation (1.5.3) by *a*_*ii*_ (the equivalence can be easily shown) expresses the tabular method as in van Arendonk et al. [9]:

(1.6) $$A_{\left(i\right)}=\left[\begin{array}{cc} A_{\left(i-1\right)} & A_{\left(i-1\right)}b_{\left(i\right)}\\ b_{\left(i\right)}^{\prime }A_{\left(i-1\right)} & a_{\mathrm{ii}} \end{array}\right]$$

Applying the partitioned matrix theory to equation (1.6), van Arendonk et al. [9] structured **A**^-1^ as a sum of *n* updates of a null matrix (recursion rule in equation 1.7) involving multiplication of a sparse vector (-**b**_(*i*)_) by itself.

(1.7) $$A_{\left(i\right)}^{-1}=\left[\begin{array}{cc} A_{\left(i-1\right)}^{-1} & 0\\ 0^{\prime } & 0 \end{array}\right]+\frac{1}{d_{\mathrm{ii}}}\left[\begin{array}{c} -b_{\left(i\right)}\\ 1 \end{array}\right]\;\left[\begin{array}{cc} -b_{\left(i\right)}^{\prime } & 1 \end{array}\right]$$

The sparse vector -**b**_(*i*)_ is actually the transpose of the *i*-th below-diagonal row of **T**^-1^ (see equation 1.3). Such a construction of **A**^-1^ requires thus to know the following:

(1) the positions and values of non-zero elements in **b**_(*i*)_, i.e. the structure of **T**^-1^;

(2) some elements of the original matrix, to compute *d*_*ii*_ as $a_{\mathrm{ii}}-b_{\left(i\right)}^{\prime }A_{\left(i-1\right)}b_{\left(i\right)}$.

After meeting these requirements (determination of the structure of the inverse triangular factor and computation of some elements of the original matrix), the same framework was extended to the inversion of other relationship matrices used in animal breeding (e.g. gametic relationship matrix [17], dominance [18] and epistasis [19] effects or covariance matrix of marked QTL effects [20]).

## Competing interests

The authors declare that they have no competing interests.

## Authors’ contributions

PF developed the method, conceived the experimental design, ran the tests and wrote the first draft. NG directed the study and made substantial contributions for the discussion. Both authors participated in writing the manuscript. All authors read and approved the final version.
